# Novel Forehead Augmentation Strategy: Forehead Depression Categorization and Calcium-Hydroxyapatite Filler Delivery after Tumescent Injection

**DOI:** 10.1097/GOX.0000000000001858

**Published:** 2018-09-06

**Authors:** Jongseo Kim

**Affiliations:** From the Kim-Jongseo Plastic Surgery Clinic, Seoul, Korea.

## Abstract

**Background:**

The minimally invasive delivery of soft-tissue calcium hydroxyapatite fillers can ameliorate the appearance of forehead depressions and wrinkles. Forehead augmentations using fillers lead to fewer adverse events and to a faster recovery time than those using surgical procedures. However, many physicians avoid using them due to the risk of pain, intravascular injections, and embolisms that can cause skin necrosis and blindness. Strategy to address these issues includes pain reduction through tumescent solution before filler injection considering the vascular pathway. In addition, the strategy is also safer from embolism.

**Methods:**

The author presents a forehead augmentation technique involving the injection of a tumescent solution for tissue hydrodissection. Not only does this create a pocket of space (TS) for filler placement, it also allows the physician to detect bleeding and prevent vessel compromise It is better to minimize the number of entry-points where no vessel exists. By minimizing the number of entry-points and physical proximity to vessels and nerves during injection, this technique reduces the risks of embolization and bleeding. For optimal, patient-customized results, the author also classifies forehead depressions in 5 different categories, depending on the severity, shape, contour, slope, and quantity of volume deficiency observed.

**Results:**

The author treated 218 patients using the author’s forehead augmentations (using one central entry-site after TS test injection). The method was easy to perform and safe from adverse effects such as vascular problem, irregularity, infection, and prolonged swelling.

**Conclusion:**

Using this simple technique, I demonstrate how calcium hydroxyapatite fillers can be injected with little force or dilution, thus preserving its original properties while increasing procedural safety, minimizing pain, restoring convexity, and creating ideal curvatures.

## Abstract 2

Supplemental Digital Content is available in the text.

## INTRODUCTION

Forehead aging, depressions, and wrinkles are caused by frontalis muscle contractions, skin aging,^1^ loss of curvature and volume, superciliary arch bulges, and forehead bossing.

Botulinum toxin improves brow elevation, relieves periorbital straining,^2,3^ and increases frontal projection.^4–6^ Calcium hydroxyapatite (CaHA) soft-tissue fillers (STF; Radiesse, RAD; Merz Pharmaceuticals GmbH, Frankfurt, Germany) provides firm^7^ and natural textures via CaHA microspheres in an aqueous carboxymethylcellulose (CMC) gel that metabolizes to calcium and phosphate ions.^8^

STF cause few adverse events^6,9^ but are avoided due to pain even though mixing with lidocaine^10–12^ or preinjection of anesthetic tumescent solutions (TS) minimizes this.^13^ I, therefore, describe TS injection hydrodissection, a simple method to facilitate low-resistance STF delivery for forehead augmentation.

## METHODS

The intervention described here can also be viewed in a video (**see video, Supplemental Digital Content 1**, which discusses the categorization of the types of depressions and contours visible frontally and laterally. This video is available in the Related Videos section of the Full-Text article at PRSGlobalOpen.com or at http://links.lww.com/PRSGO/A798).

**Video Graphic 1.**
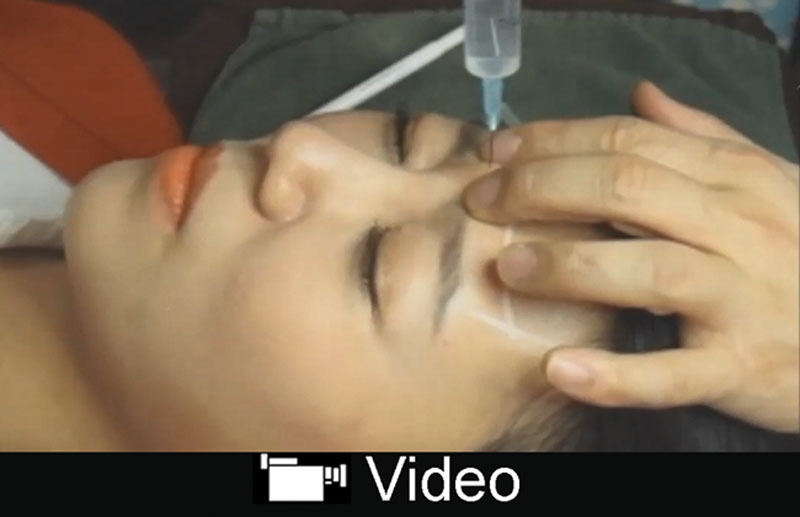
See video, Supplemental Digital Content 1, which discusses the categorization of the types of depressions and contours visible frontally and laterally. Methodology and procedures described in the article, including TS injection, hydrodissection, and filler injection are demonstrated. This video is available in the Related Videos section of the Full-Text article at PRSGlobalOpen.com or at http://links.lww.com/PRSGO/A798.

### Patients

Two hundred eighteen patients (18–48 years old; mean age, 34.7 years; 41 male, 177 female) were treated between June 2007 and June 2017. One hundred fifty-three patients were Korean, 39 were Chinese, and 26 were Japanese. Forehead depressions and contours were analyzed frontally and laterally and categorized. Type I foreheads had protruding orbital rims, and a central, localized depression toward the lower-midline and over the procerus. Type II foreheads demonstrated an above-brow triangular depression over the corrugator supercilii, with no central depression. Type III foreheads displayed a larger, centralized, and horizontally depressed area. Type IV foreheads resembled type III foreheads but contained pan-forehead depressions and a trichoma in the normal position. Type V foreheads resembled type IV foreheads but had a trichion located more posteriorly, near the lateral canthus line, and contained a pan-forehead depression.

### Materials and Tools

TS was made by mixing 0.5 mg of epinephrine and 30 cc of 2% lidocaine in intravenous bags containing 100 cc of normal saline. Anesthesia entry-points were created using insulin syringes with 29G, 1-inch long needles. 23G needles were used for cannula entry. A 40 mm long, 7 mm diameter roller (Reteenage, Seoul, Korea) evenly distributed the TS and STF. RAD was injected with 23G, 50 mm or 70 mm cannulas for soft-tissue lifting.

### Anatomical and Entry Point Considerations

**Fig. 1.**
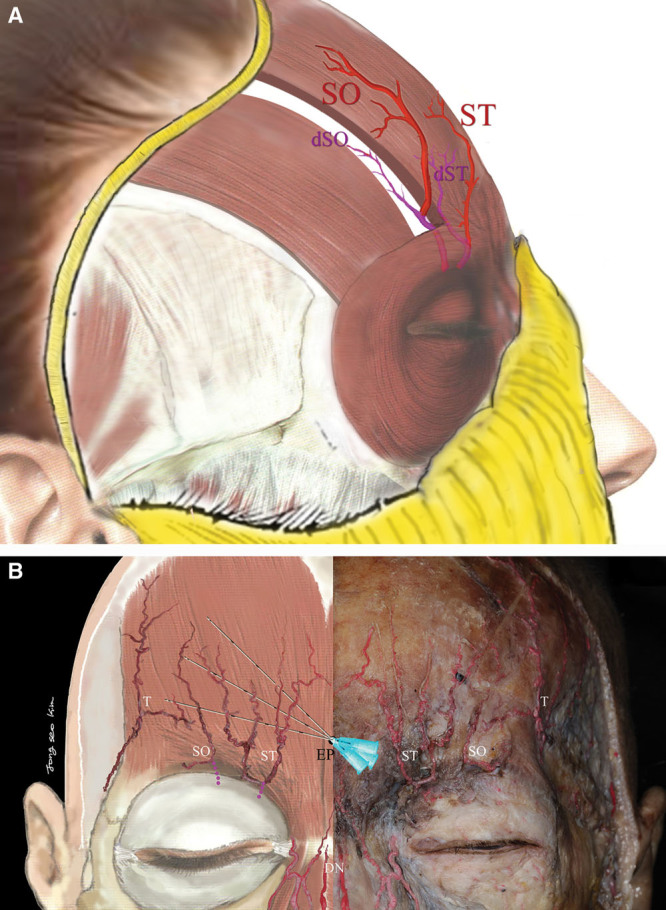
Cadaver dissection. (A) ST emerges from the lower medial margin of the orbital rim and has superficial and deep branches. Deep branch of ST (dST-purple lines) is located at the deep frontalis. The superficial branch of ST perforates the corrugator muscle and runs superficial along the anterior to frontalis muscle. The dSO and dST exit above the brow, branching periosteally before crossing the subgalea. SO emerges from the supraorbital foramen, and dSO (which might or might not exist) runs in the deep frontalis. Next, SO pierces the frontalis and then runs superficially. Therefore, the upper-part of where SO pierces the frontalis is the safest injection spot. (B) Cadaver dissection study and illustration. Fanning technique and numerous directions of needle advancement from central entry-point in forehead. Needle entry and direction of filler placement is shown. The ST and SO pierce the corrugator and frontalis and run above the muscle 1–2 cm finger breadths above the orbital rim. The ST anastomoses with the central artery, which originates from the DN, and the SO artery anastomoses with T. SO, supraorbital artery; ST, supratrochlear artery; dSO, deep branch of SO; dST, deep branch of ST; DN, dorsal nasal artery; T, superficial temporal artery; ET, entry point. Illustration by Dr. Jongseo Kim, cadaver: Courtesy of Prof. H K Kim.

Supraperiosteal and subgaleal tissue avascularity (Fig. 1A) facilitates a smooth, convex trichion-to-brow profile during augmentation. Deep supratrochlear artery (STA) branches and supraorbital (SO) arteries (SOA) are located deep to the frontalis.^14^ The STA pierces the corrugator supercilii muscle, becomes subcutaneous 15–25 mm above the SO rim,^15^ and is avoided with deep injections near the orbital rim (Fig. 1B).^16^ The lateral arteries superficial to the frontalis comprise the superficial temporal artery. The central forehead contains the central STA superficial to the frontalis (Fig. 1A, B).^17,18^ Originating from the orbital rim or superomedial orbit, 17–22 mm from the midline, the STA pierces superficially to the corrugator, deep to the orbicularis and frontalis.^19,20^ Fifteen to 25 mm above the rim, it enters these muscles and proceeds subcutaneously.^15^ At the brow, it runs vertically and 3 mm from the medial canthus. The SOA pierces the frontalis about 20–40 mm^21^ (more than 90%) above the SO rim. It may emerge intramuscularly and branch vertically through the subcutaneous tissue 15–20 mm above the rim. The SOA branches frequently to anastomose with the frontal STA at the transverse inferior and middle third forehead junction (Fig. 1B). The STA and SOA are adjacent to the bone, between the orbital rim and beneath the eyebrow, in a 1–2 cm area. Arising from the orbital rim, their cephalic portions run beneath the frontalis, around the eyebrow, superficially supramuscularly and 1–2 cm above the eyebrow. One entry-point for deep injections was created 1–2 cm above the eyebrow margin and at the facial midline, which contains fewer deep vessels.

### TS Injection and Hydrodissection

A central forehead midline entry-point was created 1–2 cm above the eyebrow (1-inch above the orbital rim) to avoid superficial STA and SOA branches. TS (1 cc) was injected with an insulin syringe through this central entry-point and distributed around the ST and SO nerves (Fig. 1B). A 23G cannula was inserted through this central entry-point and gently advanced laterally along the superperiosteal plane. During cannula advancement toward the ST and SO nerves, TS was dispensed subdermally into the left and right lateral forehead. TS was delivered by anterograde injection on both sides of the forehead for periosteum hydrodissection. TS (9 cc) was injected with over 50 passes of a 50 mm, 23G cannula inserted deep with the downward-facing bevel to create space near the frontal bone. Placement of TS lifts the vasculature above the bone and creates a separation or space for STF.

#### Entry-site Caution

Lateral hairline entry-points risk damaging large veins, while medial eyebrow entry-points risk SOA and STA embolization. Both risk commensal bacteria^22^ and biofilm^23^ contamination. These techniques require 2 entry-points; any more increases bleeding risks.

#### TS Test Injection

To dissect the galea and periosteum from the bone and create a safe space for filler placement, room temperature TS (10 cc) was injected via the midline entry-point using anterograde fanning in 50 ~ 100 strokes, between the STA, SOA, and frontal bone. Epinephrine added to TS facilitated vasoconstriction after 5 minutes and minimized bleeding. If bleeding exceeded 0.1 cc, even after 5 minutes of compression and a working assumption that the deep ST (dST) or SO (dSO) arterial branch was injured (Fig. 1), injections were postponed to avoid vascular problems and embolisms and resumed after 2 weeks. Injection of TS shifted all arteries above the TS.

### Filler Injection

The edges of a business card were aligned with the trichion and orbital rim or eyebrow to gauge the required volumization. RAD (1.5–3 cc) was dispensed into the pocket of space created by TS hydrodissection, near bone, in 50 passes using a 50 mm (or 70 mm for larger areas), 23G cannula with the bevel facing away from muscle. Specifically, 1–2 cm above the left brow, anterograde injection was initiated by placing filler at 1 mm intervals followed by 30 strokes and fanning of the cannula 90° clockwise from the horizontal (9 o’clock) to the vertical position (12 o’clock). Fillers were subsequently dispensed at the right brow, from the horizontal (3 o’clock) toward the vertical position (12 o’clock). At each stroke, 0.02–0.03 cc of RAD was injected. More convexity required more filler than the average of 1.5–3 cc. For lateral foreheads, cannulas were placed deep beneath the frontalis muscle near bone to avoid the deep SO nerves parallel to the temporal fusion line. For difficult contours, RAD was injected above this line using a bent, 70 mm long cannula. For temporal depressions, a separate entry-point was created, and additional filler was delivered.

### Injection Technique Notes

Subperiosteal injection with the periosteal-facing cannula facilitated TS hydrodissection. Cannulas are too large to inject vessels directly but can injure or tear vessel walls. Due to intravascular filler risks, injections were postponed for several weeks if test tumescent injections caused bleeding. Because filler can enter into and flow through vessels, deep forehead artery puncturing was carefully avoided. Deep injections are essential and can be ensured by touching the frontal bone with the cannula until a scratching sound is audible.

### Rolling Contouring

The treated area was massaged with a roller using moderate-high force and compression to distribute TS evenly. Five minutes postmolding, this temporary augmentation was assessed. The patient understood that the observed shape and volume would be twice that at 1-week postinjection with 3 cc RAD. After patient counseling, the volume was adjusted by ±1.5–2 cc.

### Postprocedural Care

After injection, filler was molded with a roller using low-moderate force and shaped to remove irregularities. Two hundred fifty milligrams of Ciprofloxacin, antihistamine, and Panpyrin F (300 mg acetaminophen, 18 mg methylephedrine hydrochloride, 25 mg pheniramine maleate; Dong-A Socio Holdings Co. Ltd., South Korea) was immediately administered to reduce inflammation, swelling, and tissue irritation. A cold compress was applied to the eyelid for 20 minutes in-clinic, and self-administered for 3 days thereafter. Forehead compression was avoided to prevent unintentional filler spreading and irregularities. The patient was advised that positioning the head vertically would not prevent eyelid swelling.

### Follow-up

Fine adjustments were performed after 1 week; after 3 weeks, filler integration prevents molding. Patients were advised against using headwear at 3 weeks after treatment.

## RESULTS

**Fig. 2.**
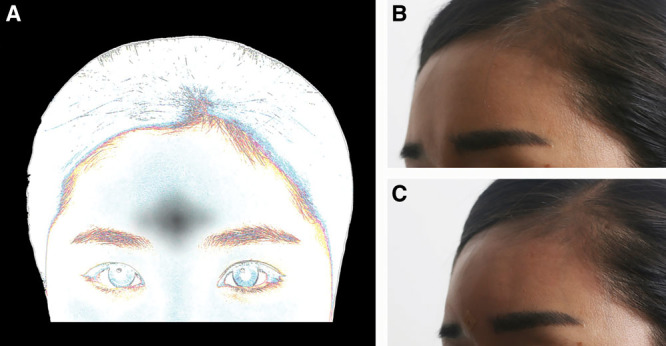
Augmentation of type I (central depression) forehead. Schematic depicting central forehead shadow (A). Patient 1 is shown before (B) and 2 days after (C) TS hydrodissection, and injection of undiluted RAD (1 cc) spread 180 degrees by tissue rolling.

**Fig. 3.**
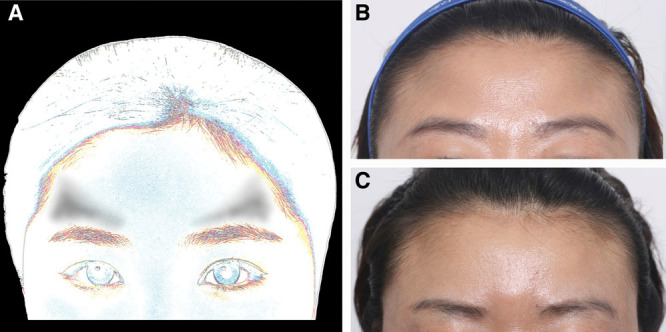
Augmentation of type II forehead (triangular depression). (A) Type II forehead in schematic showing triangular depression (A), and denoted by two gray triangles. Patient 2 before treatment (B) and 1 month (C) after receiving 1.5 cc of Radiesse.

**Fig. 4.**
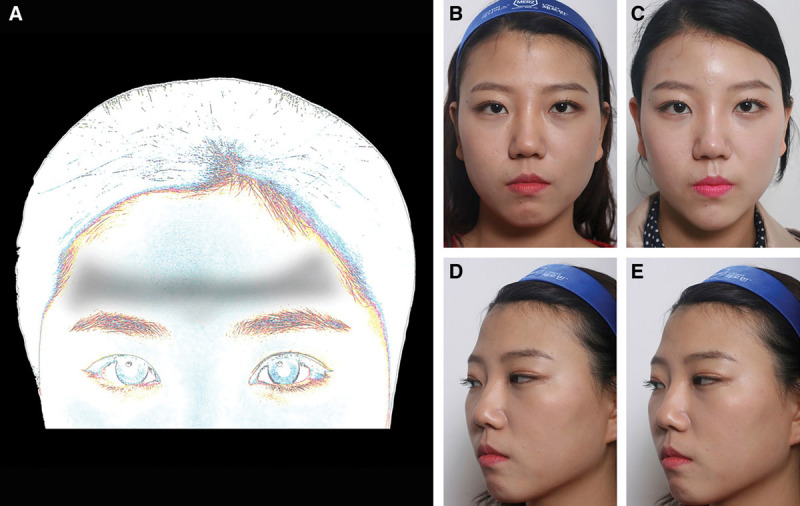
(A), RAD filler injection for forehead augmentation of type III (horizontal depression) foreheads. (B,D) Patient 3 displayed protruding hairline and eyebrows (B, D) with volume loss above the eyebrow visible as a depressed horizontal line, and at follow-up 4 days after 1.5cc of RAD filler injection (C, E).

**Fig. 5.**
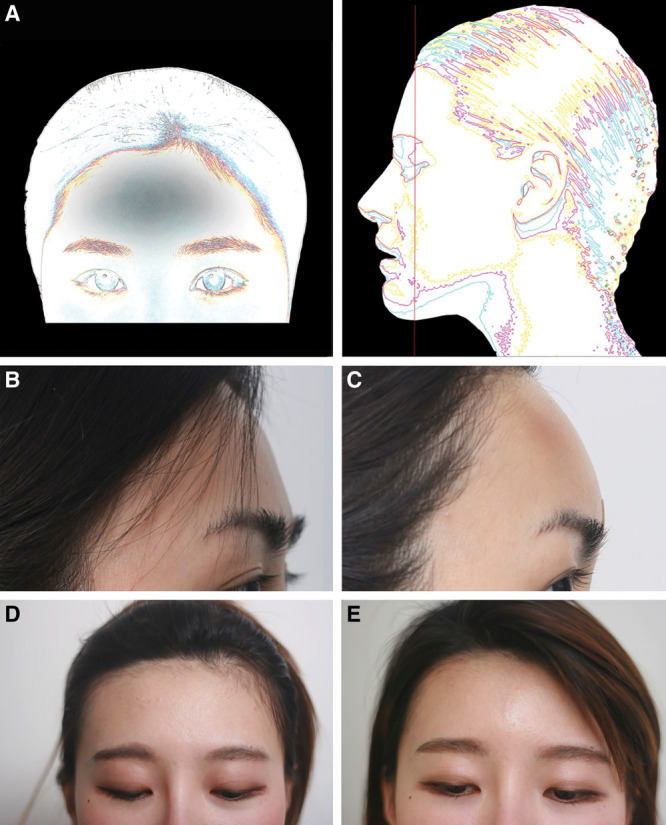
RAD filler injection for pan-forehead augmentation of type IV foreheads. (A) Schematic of patients showing all-round depression in the forehead. Patient 4 is shown at rest (B) before being injected with 6 cc of TS, followed immediately by rolling. After 5 minutes, the patient was injected with 1.5 cc of Radiesse. The day after, the forehead of Patient 4 looked bigger (as if 3 cc was injected) due to swelling and TS (C). However, it will decrease in time. Patient 5 is shown before (D) injection of 6 cc TS and 1.5 cc Radiesse, and 17 days after (E) the procedure.

**Fig. 6.**
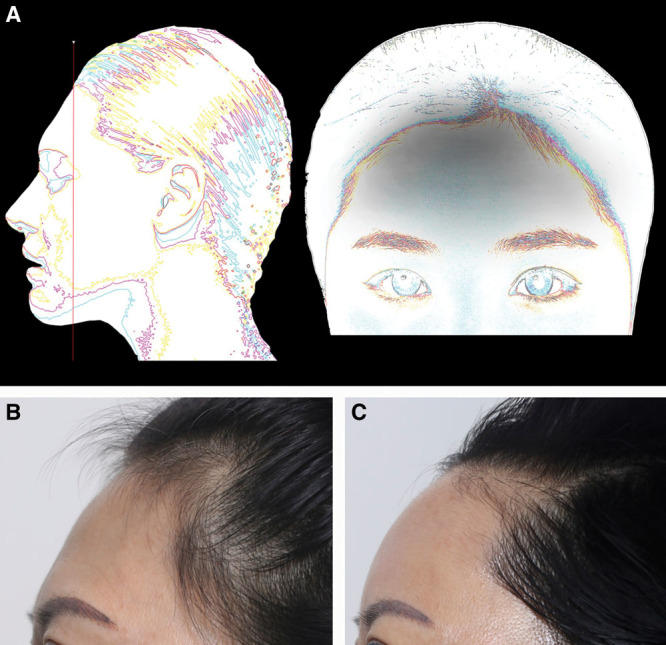
Augmentation of type V (inclined slope) forehead. Schematic of inclined slope forehead (A; trichion near horizontal level of the lateral canthus) before and after treatment. Patient 7 is shown before (B) and 3 months after injection of 4.5 cc RAD (C).

Of the patients, 69 received 1.5 cc of RAD, and 149 received 3 cc of RAD. Forehead depressions were assigned into 5 main categories. Type I foreheads (patient 1, Fig. 2) have a central, diamond-shaped depression, exaggerated glabella lines and angry appearances, requiring resolution by botulinum toxin. Type II foreheads (patient 2, Fig. 3) received augmentation of above-brow concavities as the central forehead is protruded surrounded by 2 triangular depressions. Postinjection (Fig. 3D) faces appeared smoother with kinder expressions. Type III horizontal depressions (Fig. 4) were injected horizontally and then molded. One type III patient (Fig. 4B–E) received 3 cc of undiluted RAD at (or within 1 cm of) the eyebrow. Type IV foreheads are most frequently and easily treated with an average of 3 cc of filler. As a wider forehead area is present, 1.5 cc is not always sufficient (Fig. 5), and type IV foreheads require the most filler, injected in central areas. Type V foreheads are inclined with a trichion near the lateral canthus and required filler injection more superiorly and in greater volumes (3–5 cc) than type IV foreheads (Fig. 6). Although some postprocedural swelling and residual TS would produce visible volumization, type V foreheads require the most filler, beyond the average of 3 cc. However, TS was fully absorbed 1 day postinjection.

In all cases, physician analysis of results indicated that tissue indentations were relieved, forehead projections increased, forehead curves were evened out, profiles were balanced, and skin surfaces smoothened.

Of the 69 patients receiving 1.5 cc of filler, the satisfaction rate was 100%, with patients seeking a second treatment after 6 or 12 months. Of the 149 patients receiving 3 cc of filler, the satisfaction rate was also 100%, with patients returning for a second treatment after 9–18 months.

## DISCUSSION

I describe here the categories of STF-treatable forehead depressions. The natural forehead’s differential anatomy of thin soft tissue overlying hard bone makes augmentation difficult. Traditional methods [eg, hyaluronic acid (HA) filler, fat injections, bone cement, and silicone implantation] are difficult to master, invasive, and have variable duration, pain levels, ease of correction, and exposure to carcinogens. Using HA fillers also risks the development of depressions due to the wearing of headbands. RAD is easy to deliver, molds naturally to native contours, and mimics normal textures. My novel technique for filler volumization of deficient forehead spaces provides positive patient outcomes.

Using TS hydrodissection, subperiosteal injections avoid vascular injury even near deep STA or SOA branches, which remain endangered by deep injections.^20^ The SOA deep branch is also present 1-inch above the orbital rim, and deep artery compromise can allow filler entry.

Subperiosteal injections can be performed using the described technique. However, as filler may be injected into supraperiosteal planes, especially laterally, deeper injections are necessary to avoid the deep SOA. Vein damage must be prevented, particularly around the temple or eyebrows. Medial brow injections risk embolisms due to the SOA and STA. Using my technique, filler is never injected beneath the eyebrow, but can be rolled or massaged toward this area. Midline entry points reduce the risk of vessel compromise as the area has fewer arteries and veins.

Augmentation is postponed if bleeding exceeds 0.1 cc after compression after TS test and hydrodissection injection.^1^ In my history of forehead injections, only 2 patients experienced this. Eyebrow injections must be 1 cm above the eyebrow; below this, injections must be at least 1.5 cm from danger zones. A single entry point at the bottom of the central forehead midline, 1 cm above the eyebrow margin, circumvents orbital rim arteries, which are avoided by injecting deeply. Injections over the temporal fusion line near the deep branch of the SO nerve and sensitive areas should be avoided due to risks from using the temporal approach. Medial eyebrow and lateral hairline entry-points should also be avoided due to the risk of bacterial contamination. Augmentation near the trichion is complicated by the need to inject and use pressure to spread filler there.

RAD was selected for its firmness (high G’), high elasticity, which resists pressure and deformations, and shape retention capacity. RAD is optimal for soft and harder tissue augmentations. RAD dilution is routinely practiced but decreases its viscosity and carriage of filler particles. Fillers can be compromised by handling time, temperature, and pH, and dilution with saline risks microbial or mold exposure. My technique preserves RAD’s original properties.

To minimize pain, 1.5 cc CaHA filler can be mixed with 0.3 cc lidocaine; however, there may be inadequate time for lidocaine to act. TS preinjection circumvents this, achieving pain control. To prevent severe pain and avoid injuring the deep SO nerve branch in the lateral forehead, deep injections are required.

To prevent irregularities, injections must be even, consistent, not forceful or reliant on molding, and tissue rollers should be used to smooth contours. Although rare, irregularities caused by CMC absorption can be resolved through tissue roller smoothing, as reinjection or touch-ups are not advised. Near the temporal fusion and hairlines, smooth curves must be created. Some overinjection can replace volume loss after swelling resolves. At follow-up or within 2 weeks, fine adjustments or irregularities can be addressed. After CMC absorption, neocollagenesis and fibroblast activation impede molding. Patients should avoid headwear or sleeping in a prone position that can cause indentations and depressions.

Glabellar frown line and frontalis injections can push filler upward; concurrent botulinum toxin injections can prevent this. Unlike HA fillers, particle-type fillers remain immobile. However, in the first month posttreatment, facial muscles can displace filler particles. Molding and administering botulinum toxin during follow-up may prevent filler displacement.

Procedural tissue reactions can generate interleukins and tissue factors that stimulate mast cells and platelet production, induce histamine secretion, and exacerbate inflammation after tissue injury or filler injection. Tumescent preinjection may reduce inflammation-causing immune cells, tissue swelling,^13^ anti-inflammatory cytokine (interleukin-10) production, pain, bruising, and swelling postinjection.^13,24^ TS injection also results in fewer inflammatory cells than at sites injected with filler-lidocaine mixtures.

One patient treated using a 23G cannula required a procedural halt due to bleeding. The next day, mild pain and swelling developed. Doppler assessments detected an absence of arterial blood flow and a possible embolism due to artery damage caused by filler flow through damaged vessels or decreased blood circulation due to prior neurosurgery. STA damage by cannula was found to have caused filler entry and skin necrosis.

Because the patients demonstrated here are of East Asian ethnicity, the classification system and forehead anatomic groups used are hardly transferable to an equivalent white population, because Asians are brachycephalic, whereas white populations display scaphocephaly. In Caucasians, TS would be injected via a temple entry-point using a 27G needle. The syringe would be separated from the needle, and any signs of bleeding would be evaluated. If no bleeding was observed, CaHA fillers would be injected deep (near the temporal bone) using the same 27G needle.

## CONCLUSIONS

In this simple, novel technique, TS test injections anesthetize and hydrodissect soft tissue to ease injection safe from embolism. The resulting space facilitates the gentle injection of filler, preserving its original properties, minimizing pain and nerve damage, and producing the desired facial profile.

## ACKNOWLEDGMENT

The author expresses his thanks to Dr. Shawna Tan for article writing and editorial assistance.

## Supplementary Material
